# Heavy-ion radiation-induced colitis and colorectal carcinogenesis in *Il10*^-/^^-^ mice display co-activation of β-catenin and NF-κB signaling

**DOI:** 10.1371/journal.pone.0279771

**Published:** 2022-12-30

**Authors:** Shubhankar Suman, Bo-Hyun Moon, Kamal Datta, Bhaskar V. S. Kallakury, Albert J. Fornace

**Affiliations:** 1 Department of Oncology and Lombardi Comprehensive Cancer Center, Georgetown University Medical Center, Washington, DC, United States of America; 2 Department of Biochemistry and Molecular & Cellular Biology, Georgetown University Medical Center, Washington, DC, United States of America; 3 Department of Pathology, Georgetown University Medical Center, Washington, DC, United States of America; Toho University Graduate School of Medicine, JAPAN

## Abstract

Space radiation-induced gastrointestinal (GI) cancer risk models for future interplanetary astronauts are being developed that primarily rely on quantitative animal model studies to assess radiation-quality effects of heavy-ion space radiation exposure in relation to γ-rays. While current GI-cancer risk estimation efforts are focused on sporadic GI-cancer mouse models, emerging *in-vivo* data on heavy-ion radiation-induced long-term GI-inflammation are indicative of a higher but undetermined risk of GI-inflammation associated cancers, such as colitis-associated cancer (CAC). Therefore, we aimed to assess radiation quality effects on colonic inflammation, colon cancer incidence, and associated signaling events using an *in-vivo* CAC model i.e., *Il10*^*-/-*^ mice. Male *Il10*^-/-^ mice (8–10 weeks, n = 12/group) were irradiated with either sham, γ-rays or heavy-ions (^28^Si or ^56^Fe), and histopathological assessments for colitis and CAC were conducted at 2.5 months post-exposure. qPCR analysis for inflammation associated gene transcripts (*Ptges* and *Tgfb1*), and *in-situ* staining for markers of cell-proliferation (phospho-histone H3), oncogenesis (active-β-catenin, and cyclin D1), and inflammation (phospho-p65NF-κB, iNOS, and COX2) were performed. Significantly higher colitis and CAC frequency were noted after heavy-ion exposure, relative to γ and control mice. Higher CAC incidence after heavy-ion exposure was associated with greater activation of β-catenin and NF-κB signaling marked by induced expression of common downstream inflammatory (iNOS and COX2) and pro-proliferative (Cyclin D1) targets. In summary, IR-induced colitis and CAC incidence in *Il10*^*-/-*^ mice depends on radiation quality and display co-activation of β-catenin and NF-κB signaling.

## Introduction

Epidemiological studies of A-bomb survivors have demonstrated a greater risk of gastrointestinal (GI) cancer development after low-linear energy transfer (LET) ionizing radiation (IR) such as, γ- or X-rays [1, 2]. Contrary to the established GI-cancer risk of low-LET IR, GI-cancer risk prediction for astronauts planning to travel to outer space has substantial uncertainty, mainly due to the lack of *in-vivo* data demonstrating radiation quality effects of the high-LET heavy-ion component of the space radiation [3–5]. Therefore, differential assessment of GI-cancer incidence in animal models exposed to low and high-LET radiation exposure is being conducted to understand radiation quality effects. Earlier differential assessments of GI-cancer incidence have been conducted using sporadic GI-cancer mouse models [6, 7]. Since colitis-associated cancer (CAC) constitutes a significant number of total CRC related mortality [8–10], and emerging data on heavy-ion IR-induced chronic GI inflammation [11, 12], altered microbiome [13, 14], and epithelial barrier function [15] suggest a greater but uncertain risk of colonic inflammation that might contribute to enhanced CAC incidence after heavy-ion exposure. Therefore, studies using inflammation-associated GI-cancer models are warranted to analyze the differential effect of low and high-LET radiation on CAC development.

Heavy-ion induced increased levels of pro-inflammatory factors have been attributed to the higher GI cancer incidence [11, 15, 16]. Interestingly, accumulation of pro-inflammatory factors such as cyclooxygenase (COX-1 and 2) and TGFβ1 (transforming growth factor beta-1) in the GI-tract have been reported after heavy-ion exposure [11, 17, 18], as well as during colitis and CAC development [19, 20]. Additionally, COX2 and TGFβ1 are known to activate oncogenic β-catenin signaling and NF-κB signaling, respectively and have been implicated in colitis and CAC colitis development [21, 22]. Once activated, both β-catenin and NF-kB transactivate many common target genes such as *Ptges2*, *Nos2* and *Ccnd1* and respective protein levels [COX2, inducible-NOS (iNOS) and Cyclin D1] with established roles in the development of colitis and CAC [23–27]. Furthermore, COX2 is also known to participate in a positive feedback loop leading to a vicious cycle of continuous β-catenin activation and CAC development [28].

The *Il10*^-/-^ mouse is a well-characterized mouse model to study the progression of CAC and recapitulate progressive colonic inflammation leading to CAC, as observed in humans [29–33]. In this study, using *Il10*^-/-^ mice, we demonstrate that exposure to heavy-ion radiation is associated with a higher incidence of colitis and CRC as well as co-activation of β-Catenin and NF-κB signaling. Additionally, this study also emphasizes that colitis and CAC incidence in *Il10*^-/-^ mice are dependent on radiation quality which has implications for understanding space radiation-induced CAC and overall CRC risk among astronauts.

## Materials and methods

### Mouse breeding, genotyping and maintenance

Male and female C57BL6/J mice (stock# 000664) were bred and maintained at the Georgetown University (GU) animal facility. Female *Il10*^-/-^ mice (stock#002251) were purchased from Jackson Laboratory (Bar Harbor, ME), and crossed with wild-type male C57BL6/J mice to obtain heterozygous (*Il10*^+/-^) male and female mice. Further, heterozygous males and females were bred and genotyped to obtain *Il10*^-/-^ mice. Genotyping was performed using tail DNA and triple primer PCR assay 1. Mutant reverse (5’ CCACACGCGTCACCTTAATA 3’); 2. Common forward (5’ CTTGCACTACCAAAGCCACA 3’) and 3. Wild-type reverse (5’ GTTATTGTCTTCCCGGCTGT 3’). A 20 μL PCR reaction was set up using 2x PCR master mix, 1 μL tail DNA, 0.5 μM primer with thermocycler settings of 94°C for 5 min followed by 40 cycles of (94°C for 15 sec., 60°C for 15 sec. and 72°C for 15 sec.) and 72°C for 2 min. After weaning, male *Il10*^-/-^ mice were group-housed (5/cage) at the GU animal facility with easy access to food and water in a light cycle (12h light/dark), temperature and humidity (50%) controlled environment. At 8–10 weeks after birth, genotyped male *Il10*^-/-^ mice (n = 12/group) were randomly assigned to either sham, γ-rays, and heavy-ion (^56^Fe or ^28^Si) groups. All animal maintenance and experimental procedures including irradiation, euthanasia, and sample collection were performed in accordance with the approved IACUC protocol. All research personnel involved in direct animal handling completed their animal training prior to any contact with animals.

### Irradiations

The γ-ray exposure was done using a ^157^Cs-irradiator, while heavy-ion (^28^Si and ^56^Fe) exposures using previously determined γ-ray 2 Gy equitoxic doses i.e. 1.6 Gy of ^56^Fe and 1.4 Gy of ^28^Si 1.4 Gy were done at the NASA space radiation laboratory (NSRL), in Brookhaven National Laboratories (BNL) [34, 35]. S1 File describes the γ-ray equitoxic dose calculation for ^56^Fe and ^28^Si radiation. Briefly, animals were exposed to γ (2 Gy), ^56^Fe (1.6 Gy; 148 keV/μm) or ^28^Si (1.4 Gy; 69 keV/μm), and then all animals were housed and regularly monitored throughout the study period at Georgetown University (GU) animal facility. All experimental animals were subjected to similar housing conditions and any adverse health issues were promptly addressed as per our approved animal protocols at GU and BNL. The GU-IACUC protocol # 07–009 2022 (renumbered as # 2016–1129) was initially approved in 2007 and was renewed every three years with the current expiration date of Dec. 10, 2024, and the BNL-IACUC protocol # 345, was initially approved in 2007 and renewed annually with the current expiration date of Feb. 6, 2023. During the post-radiation follow-up period all animals were monitored twice daily for sign of discomfort and distress including reduced activity, hunched posture, diarrhea, and weight loss (>15% relative to cage mates). Any mouse with declining health were euthanized by CO_2_ asphyxiation within 4 to 6 hours of notice and was excluded from the study.

### Histopathology

Mice were placed in a carbon dioxide chamber to euthanize and colon tissues were resected for swiss-rolls preparation. Formalin-fixed colon swiss-rolls were sectioned and stained with hematoxylin and eosin (H&E) for histological examination of colitis and CAC. A board-certified pathologist, blind to the experimental groups, analyzed the sections for colitis and tumor grade in control and all irradiated groups. For qualitative assessment, colon inflammation was noted on a scale of 0 to 4, where 0 represents normal mucosa; 1. mild epithelial inflammation; 2. noticeable inflammation with intact mucosa; 3. inflammations with mucosal swelling; and 4. severe colitis with structural damage. Colon tumors were classified as either adenoma or invasive carcinoma marked by epithelial hyperplasia or invasion of the sub-mucosal compartments, respectively.

### Immunohistochemistry

After the deparaffinization and rehydration step, colonic swiss roll sections were subjected to antigen retrieval using 1x citrate buffer solution (pH 6.0, Invitrogen, Carlsbad, CA) in a microwave for 15 min. After peroxidase and protein blocking steps, sections were subjected to overnight incubation at 4°C with respective primary antibodies i.e. active β-catenin (Cat#05–665, Millipore, Billerica, MA); cyclin D1 (Cat # 04–1151, Millipore); phospho (Ser311) NF-κB p65 (PA5-97363, ThermoFisher Scientific, Waltham, MA); iNOS (Cat#ab15323, Abcam, Cambridge, MA); COX2 (Cat#12282, Cell Signaling Technology, Danvers, MA), and phospho(Ser-10)-histone H3 (Cat# 09–797; Millipore). Finally, all slides were processed using a IHC detection kit (Cat # ab236466, Abcam), as per manufacturer’s instruction, and hematoxylin was used as a counterstain. All images were acquired using a bright field microscope and image quantification was done using Fiji (Image J) software package [36]. A total of 8 to 10 digitalized image/group was quantified and data are presented either as DAB intensity or number/high power field (HPF).

### mRNA expression analysis

Normal and tumor tissues from formalin-fixed paraffin-embedded (FFPE) blocks were micro-dissected with the help of H&E-stained serial sections. Total RNA from both normal and tumor tissue regions of FFPE blocks were isolated using the RNeasy FFPE Kit (Cat#73504, Qiagen, Germantown, MD). Finally, 2μg of total RNA was used for cDNA preparation using RT^2^ first strand cDNA synthesis kit (Cat#330404, Qiagen). And qPCR reaction was set up using respective forward and reverse primers for *Ptges1 [Forward (*5’TACAGGAACTCCACTGGTG3’*; Reverse* 5’AAAGCCCAGAATTCCTCCC3’*]*, *Ptges2 [Forward* 5’CAAGTACTGGCTCATGCTG3’*; Reverse* 5’ CACTTCATCTCCTCCGTCC3’*]*, *Ccnd1 [Forward* 5’AGACCATTCCCTTGACTGC3’*; Reverse* 5’AAGCAGTTCCATTTGCAGC3’*]*, *Nos2 [Forward* 5’ CCAACAATACAAGATGACCCT3’*; Reverse* 5’TTCTGGAACATTCTGTGCTG3’*]*, *Tgfb1[Forward* 5’ACCAAGGAGACGGAATACAG3’*; Reverse* 5’CGTTGATTTCCACGTGGAG3’*]*, and *Actb [Forward* 5’GACTCCCTTCTATGAGCTGAG3’*; Reverse* 5’GAAGGTCTCAAACATGATCTCG3’*]* using SsoAdvanced™ universal SYBR green supermix (Cat#1725271, Bio-Rad, Hercules, CA) as per manufactures recommendations. Finally, mRNA expression was calculated using the comparative CT method using *Actb* as a housekeeping gene for normalization.

### Statistical analysis

For quantitative analysis of colitis score, tumor number, and carcinoma count, equality of variance was determined using non-parametric Levene’s test with sample size (n = 12 mice/group), and statistical significance (p<0.05) between control and irradiated groups were determined using Welch’s one-way ANOVA (analysis of variance) followed by a post hoc test. In the case of IHC and qPCR analysis, statistical significance (p<0.05) was determined using a two-tailed paired student’s t-test. All statistical analysis was performed using GraphPad Prism software (La Jolla, CA).

## Results

### Inflammatory changes in C57BL6 mouse colon after γ and heavy-ion irradiation

**Fig 1:**
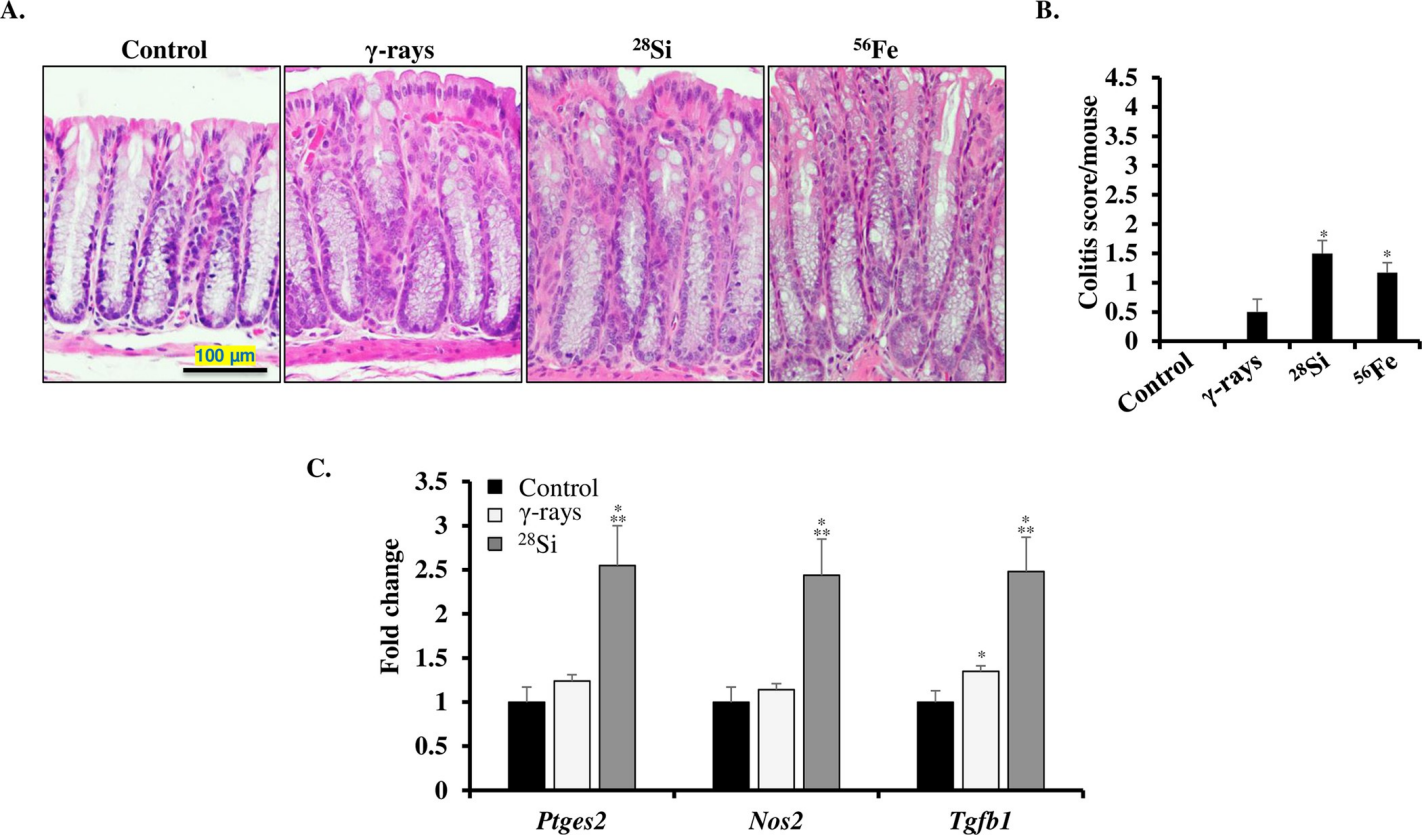
Heavy-ion exposure induced inflammatory changes in wild-type mice colon. A) Representative H&E-stained images of colonic mucosa. B) Induction of mild colitis (score 1 to 1.5) after exposure to γ (2 Gy) equitoxic doses of, ^28^Si (1.4 Gy), and ^56^Fe (1.6 Gy) at 2 months post-exposure. Colitis was scored on a scale of 0 to 4 and the average colitis score has been presented as a bar graph. C) Fold change in expression of inflammation-associated genes (*Ptges2*, *Nos2*, and *Tgfβ1*) in irradiated mice colon at 2 months post-exposure. All bars show mean ± SEM and * indicates a p-value <0.05 compared to control and ** indicates p<0.05 compared to the γ exposed group.

Mild epithelial inflammation was noted in heavy-ion (^28^Si and ^56^Fe) irradiated wild-type mouse colon samples (**Fig 1A**). Quantitative histopathological assessment indicated the signs of mild colitis (score between 1 and 1.5) in segments of heavy-ion irradiated mouse colon (**Fig 1B**). In comparison to the control group, no statistically significant increase in colitis score was observed after 2 Gy γ-ray exposure at 2 months post-exposure. The significantly higher expression of pro-inflammatory genes (*Ptges2*, *Nos2*, and *Tgfb1*) in the ^28^Si-irradiated mouse colon compared to the γ, and control groups supported the histological findings of mild colonic inflammation after ^28^Si exposure (**Fig 1C**). However, despite mild colonic inflammation, and augmented expression of inflammatory genes, no incidence of CAC was noted in wild-type mice, therefore, further studies to explore the association between heavy-ion induced colonic inflammation and CAC were conducted using *Il10*^*-/-*^ mice.

### Increased colitis and CAC in heavy-ion irradiated Il10^-/-^ mice

**Fig 2:**
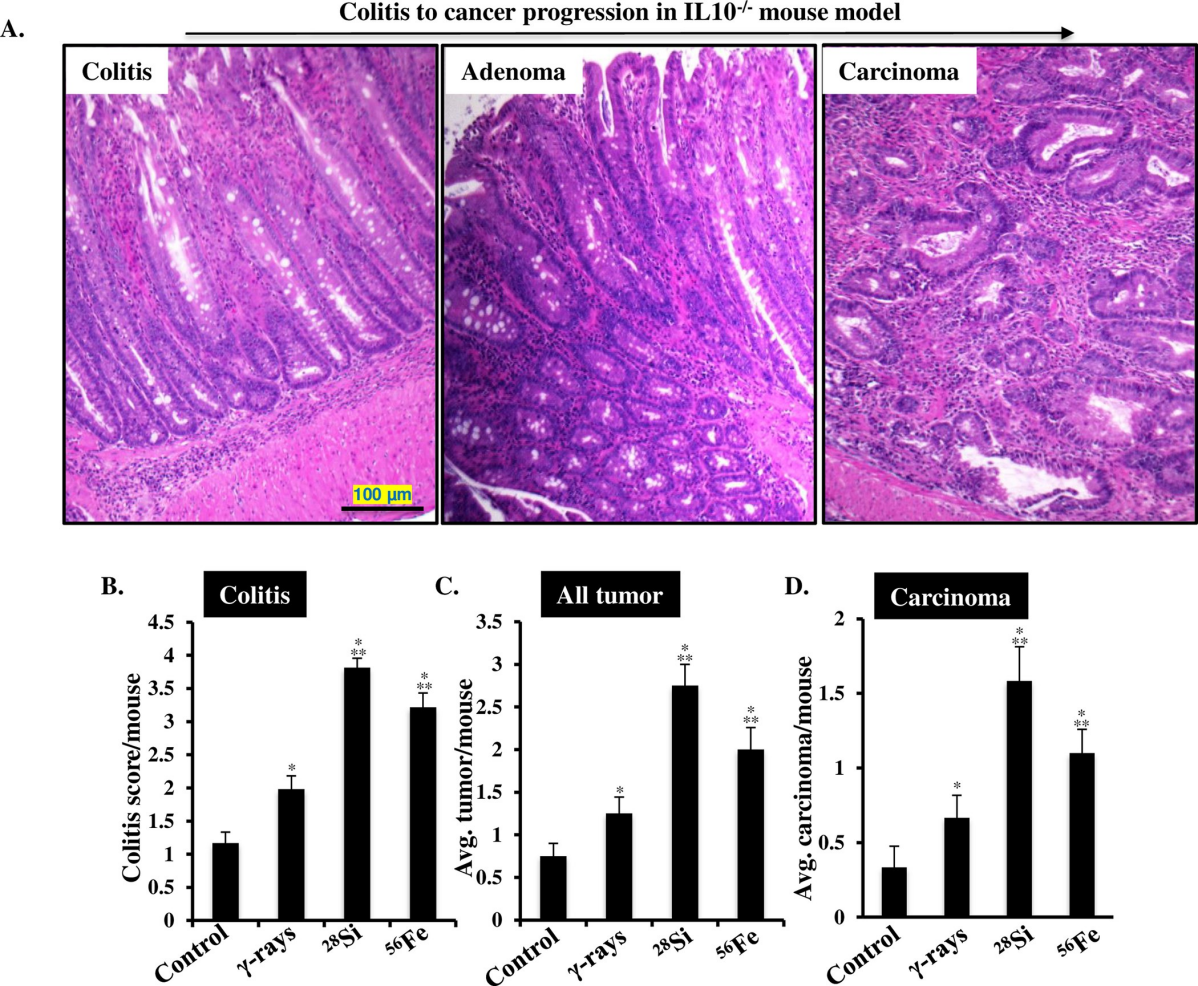
Heavy-ion exposure induced colitis and colon cancer in *Il10*^-/-^ mice. A) Representative H&E-stained images (100X magnification) of colitis, adenoma, and carcinoma in *Il10*^-/-^ mice. B) Induction of colitis after exposure to equitoxic doses of γ (2 Gy), ^28^Si (1.4 Gy), and ^56^Fe (1.6 Gy) at 2.5 months post-exposure. Colitis was scored on a scale of 0 to 4 and the average colitis score has been presented as a bar graph. C) The average number of colonic tumors at 2.5 months after γ, ^28^Si, and ^56^Fe exposure. D) The average number of carcinomas at 2.5 months after γ, ^28^Si, and ^56^Fe exposure. All bars show mean ± SEM and * indicates a p-value <0.05 compared to control and ** indicates p<0.05 compared to the γ exposed group.

*Il10*^*-/-*^ mice displayed progression of colonic inflammation to colon cancer as observed in human CAC development (**Fig 2A).** Heavy-ion irradiation caused a significantly higher increase in colonic inflammation, tumor, and carcinoma incidence relative to γ radiation in *Il10*^-/-^ mice. The average colitis score in the control group was 1.17±0.17, and the highest colitis score was observed in ^28^Si exposed mice (3.81±0.14) followed by ^56^Fe (3.2±0.22) and γ (1.98±0.2) (**Fig 2B**). Colitis scores in both ^28^Si and ^56^Fe exposed mice colon were significantly (p <0.05) higher compared to γ-exposed mice. Further, the average number of total tumor (adenoma and carcinoma) count per mouse in the control group was 0.75±0.25, and the highest tumor frequency was observed in ^28^Si exposed mice (2.75±0.25) followed by ^56^Fe (2.0±0.37) and γ (1.25±0.39) (**Fig 2C**). Mean tumor number after ^28^Si and ^56^Fe exposure was significantly (<0.05) higher compared to γ irradiated mice. In addition, the number of carcinomas per mouse in the control group was 0.33±0.14, and the highest carcinoma frequency was observed in ^28^Si irradiated mice (1.58±0.23), followed by ^56^Fe (1.08±0.26) and γ (0.66±0.21). The average number of carcinomas per mouse after ^28^Si and ^56^Fe exposure was significantly (p <0.05) higher compared to γ irradiated mice (**Fig 2D**).

### Heavy-ion radiation-induced colitis and CAC display upregulated expression of pro-inflammatory genes accompanied by increased cell proliferation

**Fig 3:**
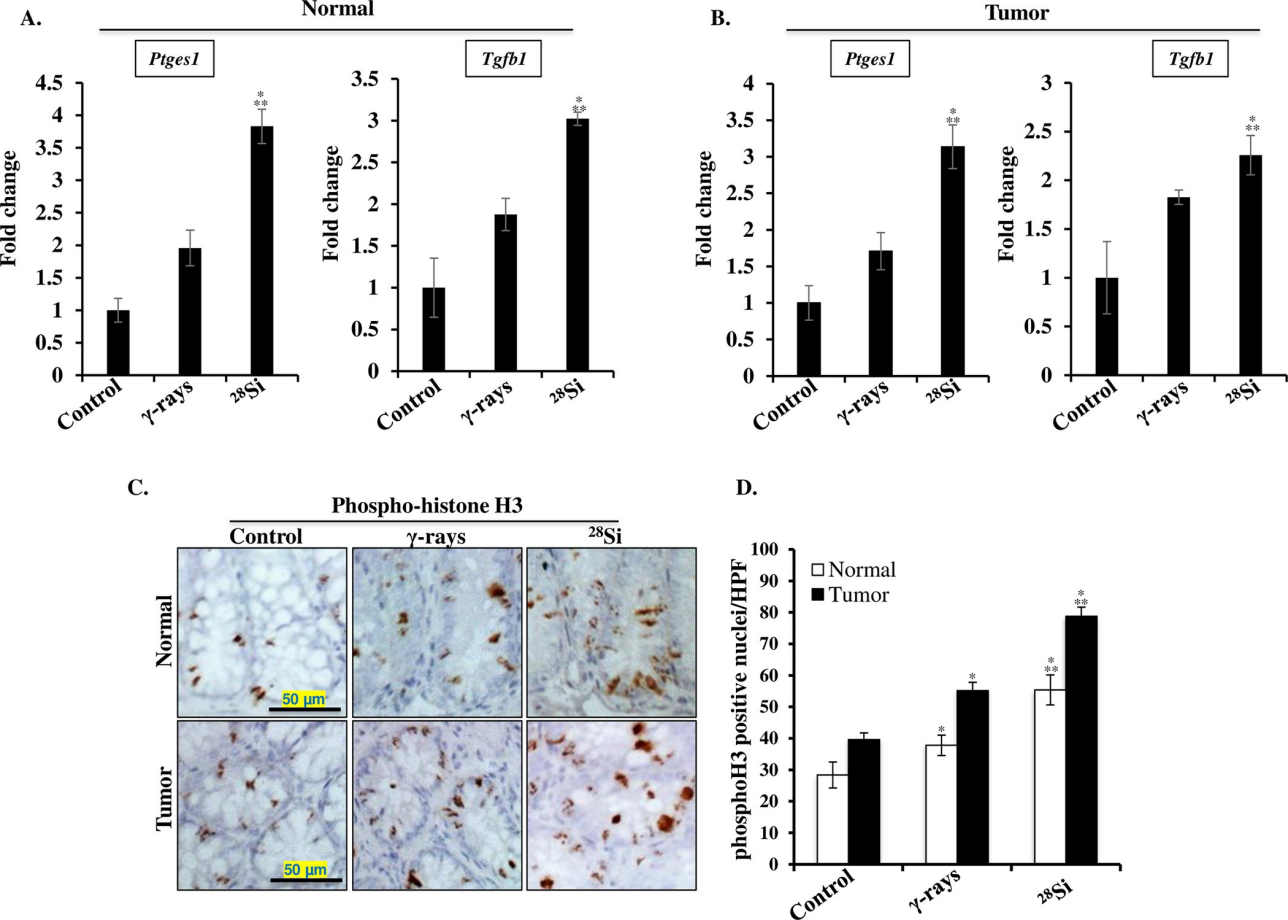
Greater increase in pro-inflammatory gene expression and mitotic cell population in the colonic tissues of the heavy-ion exposed mice. A) Fold change in expression of inflammation-associated genes (*Ptges1* and *Tgfβ1*) in normal colon. B) Fold change in expression of *Ptges1* and *Tgfβ1* in colon tumor. C) Representative images (200X magnification) of phospho-H3 (mitotic cells) stained tumor-free (normal) and tumor region from control, γ-rays, and ^28^Si irradiated mice. D) Quantification of pH3 positive nuclei per high-powered microscopic field (HPF) in colonic normal and cancer tissues. All bars show mean ± SEM and * indicates a p-value <0.05 compared to control and ** indicates p<0.05 compared to the γ exposed group.

Among ^28^Si- and ^56^Fe-ion irradiated mice, the colitis score and CAC incidence were higher in ^28^Si exposed mice, therefore follow-up molecular analysis and differential changes in pro-inflammatory and oncogenic signaling were assessed in the colon tissues obtained from control, γ, and ^28^Si-ion irradiated mice. The mRNA expression analysis of inflammation-associated genes in the normal colon and CAC samples revealed a differential expression of *Ptges1* and *Tgfb1* genes in control, γ and ^28^Si-ion exposed mice, where *Ptges1* and *Tgfb1* gene expression was significantly higher in ^28^Si irradiated mice, relative to γ rays. (**Fig 3A and 3B**). Further, quantitative analysis of a mitotic marker (phospho-histone H3) immuno-stained sections showed a higher number of proliferating cells in both normal mucosa and CAC of the heavy-ion irradiated mice, compared to the control and γ exposed groups (**Fig 3C and 3D**).

### Heavy-ion irradiation led to co-activation of β-catenin and NF-κB signaling

**Fig 4:**
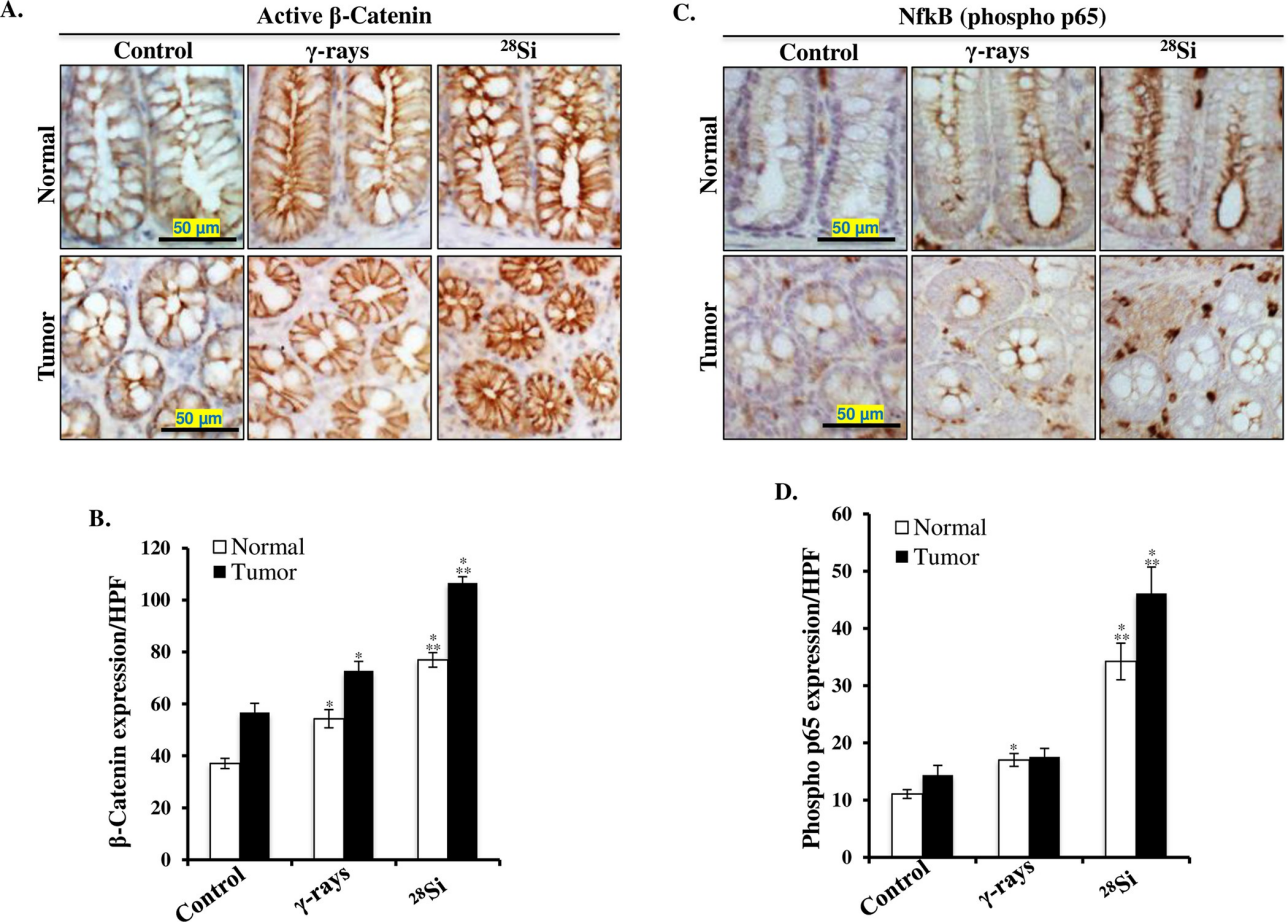
Increased activation of β-catenin and NF-κB in heavy-ion exposed mouse colon. A) Representative images (200X) of active β-catenin-stained tumor-free (normal) and tumors from control, γ-rays, and ^28^Si irradiated mice. B) Quantification of active β-catenin staining measured as DAB intensity per high-powered microscopic field (HPF) in normal and cancer tissues. C) Representative images (200X) of phospho-p65 (NF-κB) stained tumor-free (normal) and tumors from control, γ-rays, and ^28^Si irradiated mice. D) Quantification of phospho-p65 staining measured as DAB intensity per HPF in normal and tumor tissues. All bars show mean ± SEM and * indicates a p value <0.05 compared to control and ** indicates p<0.05 compared to the γ exposed group.

**Fig 5:**
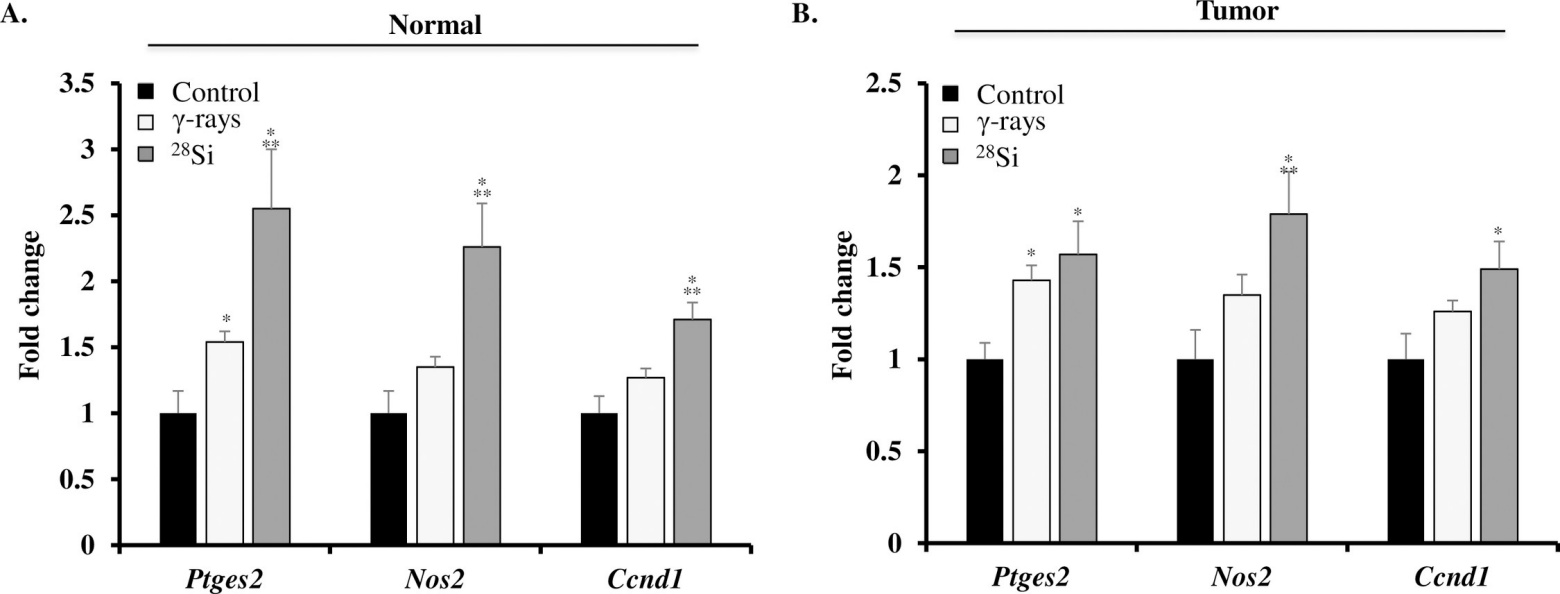
Increased expression of β-catenin and NF-κB target genes in heavy-ion exposed mouse colon. A) Fold change in expression of β-catenin and NF-κB target genes *(Ptges2*, *Nos2*, and *Ccnd1)* in normal colonic mucosa. B) Fold change in expression of β-catenin and NF-κB target genes *(Ptges2*, *Nos2*, and *Ccnd1)* in colon tumor. All bars show mean ± SEM and * indicates a p <0.05 compared to control and ** indicates p<0.05 compared to the γ exposed group.

**Fig 6:**
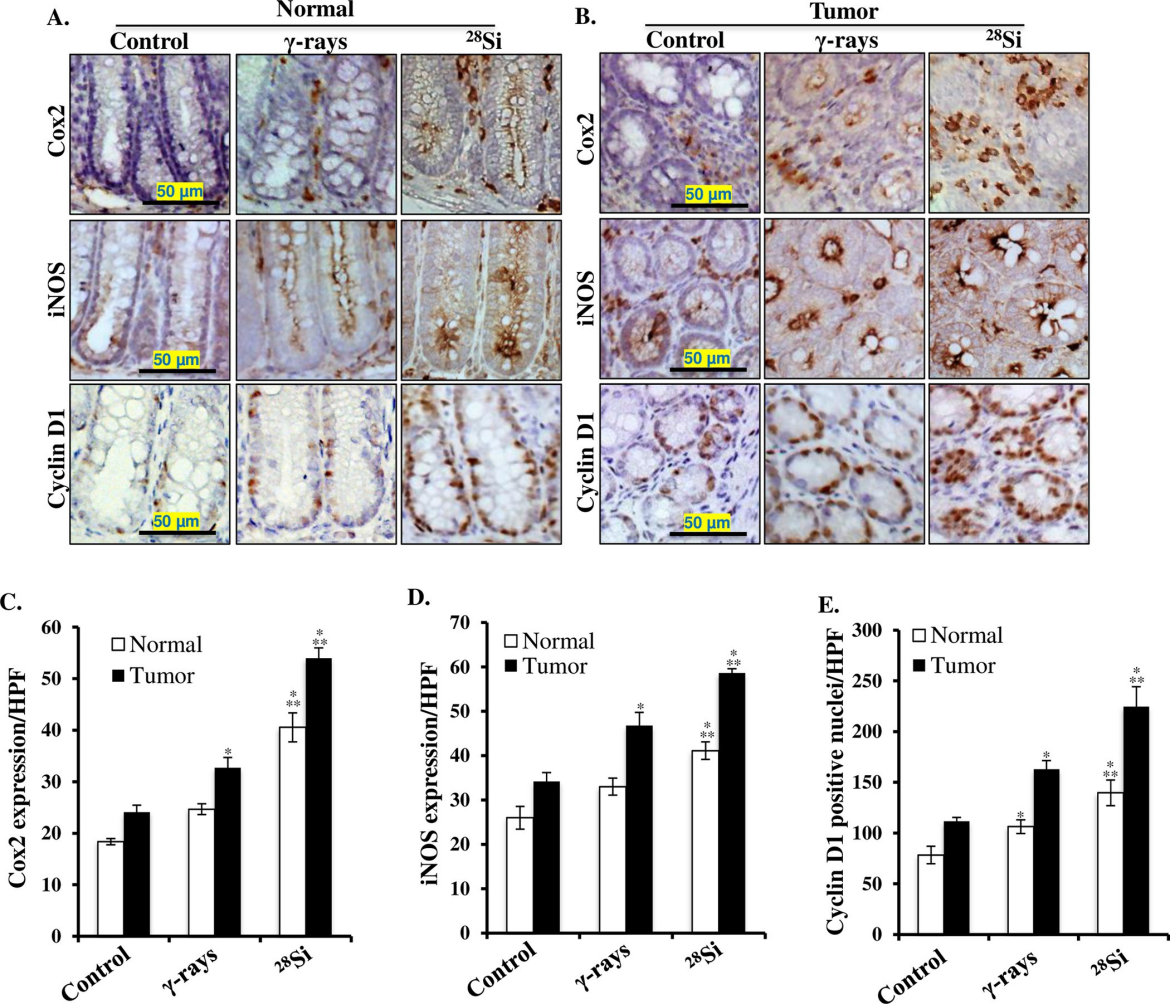
Greater accumulation of common transcription targets of β-catenin and NF-κB with known pro-inflammatory and pro-proliferative functions in heavy-ion exposed mouse colon. A) Representative images (200X) of COX2, iNOS, and cyclin D1 stained tumor-free (normal) colon sections from control, γ-rays, and ^28^Si irradiated mice. B) Representative images (200X) of COX2, iNOS, and cyclin D1 stained colon tumor from control, γ-rays, and ^28^Si irradiated mice. C) Quantification of COX2 protein expression measured as DAB intensity per high-powered microscopic field (HPF) in normal and cancer tissues. D) Quantification of iNOS protein expression measured as DAB intensity per HPF in normal and cancer tissues. E) Quantification of cyclin-D1 positive nuclei per HPF in normal and cancer tissues. All bars show mean ± SEM and * indicates a p <0.05 compared to control and ** indicates p<0.05 compared to the γ exposed group.

Immunohistochemically stained colon tissue sections from sham, γ, and ^28^Si exposed mice showed significantly higher expression of active β-catenin in both the tumor areas and tumor-free normal mucosa (**Fig 4A**). Quantification of DAB signal showed significantly higher staining in ^28^Si exposed mice relative to control and γ radiation groups (**Fig 4B**). Immunohistochemically stained colon tissue sections from sham, γ, and ^28^Si exposed mice showed significantly higher expression of phspho-p65 (active subunit of NF-κB) in both the tumor areas and tumor-free normal mucosa (**Fig 3C**). Quantification of the DAB (brown chromogen) signals showed significantly higher staining for phspho-p65 in ^28^Si exposed mice relative to control and γ radiation groups (**Fig 4D**). Further, qPCR analysis of normal appearing colonic mucosa and tumor samples showed that the expression of β-catenin and NF-κB downstream gene targets *(Ptges2*, *Nos2*, and *Ccnd1)* were greater in ^28^Si exposed mice relative to control and γ radiation groups (**Fig 5A** and **5B**). Additionally, immunohistochemical staining for β-catenin and NF-κB downstream targets at protein level i.e., COX2 (encoded by *Ptges2*), iNOS (encoded by *Nos2*), and cyclinD1 (encoded by *Ccnd1*) showed higher expression in both tumors as well as normal colonic mucosa after heavy ion exposure, relative to control and γ radiation (**Fig 6A and 6B**). Quantitative analysis of COX2, iNOS, and cyclin D1 expression from multiple frames of acquired images showed significantly higher expression in normal mucosa (p<0.05) as well as in tumor area (p<0.05) compared to both control and γ exposed mice (**Fig 6C–6E**).

## Discussion

**Fig 7:**
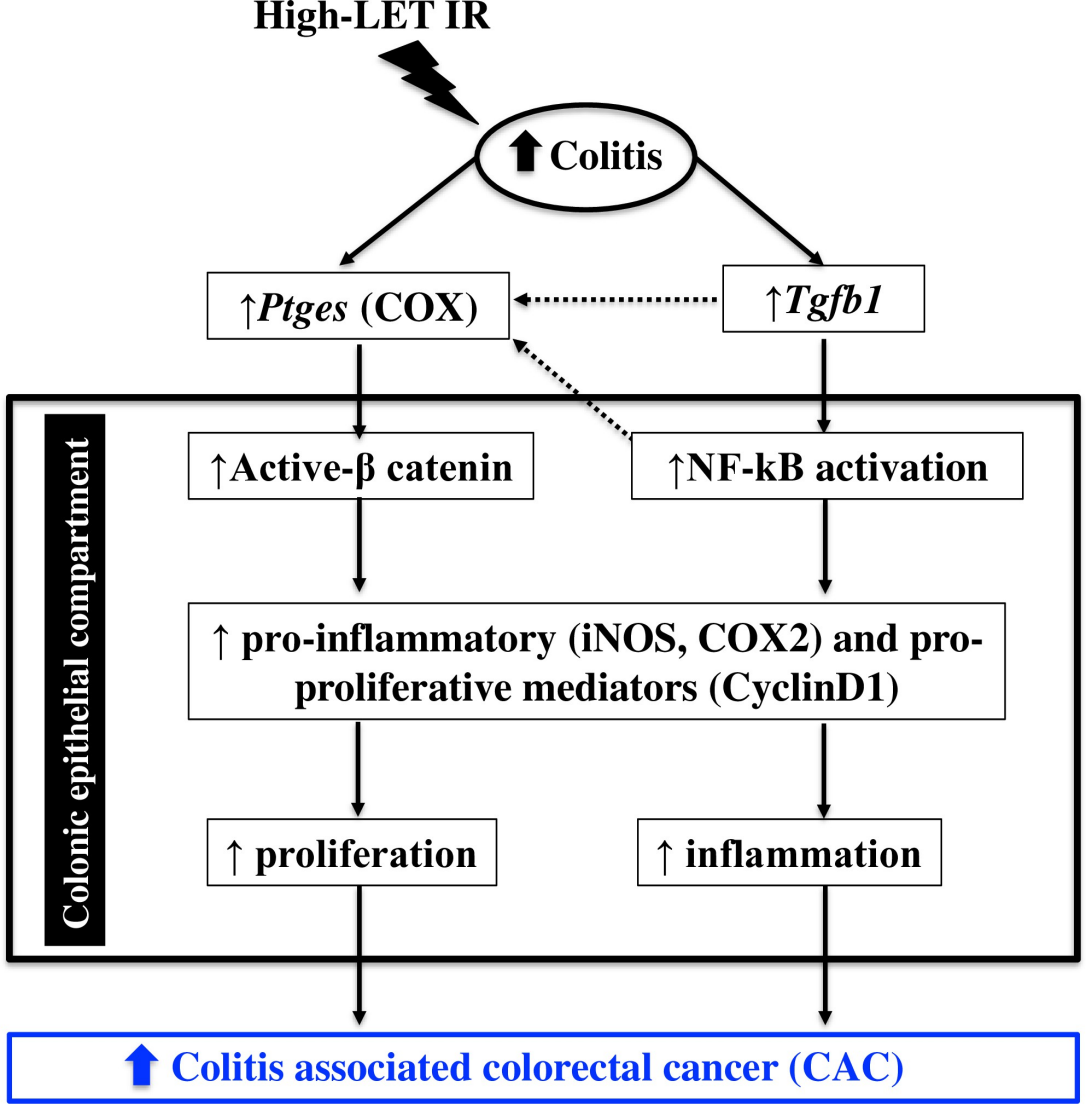
An illustrative summary of signaling events associated with heavy-ion radiation-induced colitis-associated colorectal cancer (CAC).

Assessment of qualitative and quantitative differences in carcinogenic incidence in surrogate mouse models after low and high-LET radiation exposure is a well-accepted approach to understanding radiation quality effects. In this study, using a mouse model of human CAC, we have demonstrated that exposure to high-LET heavy-ions (^28^Si and ^56^Fe) resulted in an accelerated colitis, increased tumor number, and carcinoma count. We also showed that after irradiation both normal-appearing colonic mucosa and tumor display differential co-activation of β-catenin and NF-κB signaling that was much higher in heavy-ion exposed mice, relative to γ-rays (**Fig 7**). Accordingly, higher expression of pro-inflammatory (COX2, and iNOS) and pro-proliferative (cyclin D1) downstream effectors were also evident in heavy-ion exposed mice.

Histopathological and molecular analysis of IR-induced colitis and CAC in *Il10*^-/-^ mice displayed a dependence on radiation quality with the highest effect after ^28^Si irradiation (LET 69 keV/micron). A similar pattern of LET-dependent GI-tumorigenesis has also been observed in adenomatous polyposis coli (APC) gene-based sporadic GI-cancer mouse (*Apc*^*min/+*^ and *Apc*^*1638N/+*^) models [7, 37], therefore, this study complements our earlier studies and also emphasizes the *Apc*-gene independent aspect of GI-tumorigenesis after low- and high-LET IR exposure. Additionally, the similar trend in radiation quality-dependent GI-tumorigenesis in *Il10*^-/-^ mice suggests a genotype-independent tumorigenic effect of heavy-ion radiation and also supports the notion that the carcinogenic effect of heavy-ion radiation is greater than low-LET γ-rays.

In the context of space exploration, the heavy-ion radiation doses and dose rate used in this study are high yet demonstrates a radiation quality-dependent increase in colitis and CAC incidence. Moreover, the relative biological effectiveness (RBE) values determined in the spontaneous GI-tumorigenesis mouse model i.e., *Apc*^1638N/+^ mice at a 10 to 200 cGy dose range of heavy-ion radiation have indicated an inverse dose relationship with a higher RBE at lower doses [6]. Therefore, additional studies are required using a space-relevant dose range to assess the RBE values for colitis and CAC model. Higher RBE of heavy-ions for cancer development have been attributed to a greater non-targeted effects, including bystander effects [38], where bystander effect represents the transmission of oncogenic signals from a directly hit cell to a nearby cell, and the role of pro-inflammatory mediators such as TGFβ1, iNOS, and COX2 in IR-induced bystander effects is well documented [39, 40]. Notably, higher expression of TGFβ1, iNOS, and COX2 (*Ptges*) is also associated with colitis and CAC incidence [41–43]. Increased COX2 and its pro-inflammatory byproduct PGE2 (prostaglandin E2) in the GI-tract have been observed after heavy-ion radiation exposure induced GI-tumorigenesis [11, 12]. Increased iNOS expression also indicates the involvement of nitrosative stress in heavy-ion induced colitis and CAC development, and also is consistent with earlier reports of increased nitric oxide and nitrosative damage in the mouse GI-tract after heavy-ion irradiation [35].

COX2 byproduct prostaglandin-E2 (PGE2) and TGFβ1 are known to activate oncogenic β-catenin signaling and NF-κB signaling, respectively and both have been independently implicated in CAC and colitis development [21, 22]. Heavy-ion irradiation resulted in increased colitis and CAC incidence, where higher co-activation and complementarity between β-catenin and NF-κB signaling were evident. The crosstalk between these two important signaling networks have also been reported to complement each other during CAC pathogenesis [22, 44–46]. The positive regulation of NF-κB activity by β-catenin has been reported through differential regulation of the NF-κB target genes [47], whereas β-catenin knockdown has been shown to reduce NF-κB transcriptional activity [48]. Moreover, differential functional regulation of their common protein targets (COX2, iNOS, and Cyclin D1) has also been attributed to direct physical interactions between β-catenin and NF-κB [49].

Interleukin-10 (IL10) has an established anti-inflammatory role in GI tissues and polymorphisms in the *Il10* gene are often associated with higher CRC risk [50–52]. Moreover, an association between *Il10* polymorphism and IR-induced differential GI-cancer incidence has also been noted in A-bomb survivors [53]. Analogous to the colitis and CAC incidence in humans, GI-pathogenesis in *Il10*^*-/-*^ mice is dependent on environmental factors and follow a multi-hit model of carcinogenesis [32], that may involve colonic epithelial and other cell types, such as immune cells. IR-induced chronic epithelial inflammation is known to include persistent oxidative stress, reduced autophagy, altered cellular differentiation, perturbed mucosal cell physiology depicting an accelerated aging phenotype, and increased senescence-inflammatory response (SIR) resulting in increased expression of pro-inflammatory mediators [11, 15, 16, 54–56], however the effect of heavy-ion on colonic immune cells is largely unknown and future studies exploring the role of immune cells in the onset of colitis and immune-epithelial cell interaction in the development of heavy-ion induced CAC is required.

In summary, our study demonstrated that exposure to heavy-ion radiation (^28^Si or ^56^Fe) is associated with a greater incidence of colonic inflammation, colitis, and CAC in *Il10*^-/-^ mice (**Fig 7**). Molecular analysis suggested that relative to γ rays, heavy ions caused higher activation of β-catenin and NF-κB signaling associated with higher pro-inflammatory and proliferative responses in both normal and tumor region of the mouse colon. Analysis of heavy-ion exposure associated increase in colitis and CAC incidence has implications in understanding CAC risk for deep space astronauts and also for devising chemopreventive strategies to minimize CAC incidence and overall colorectal cancer risk among astronauts. Moreover, this study should also help in understanding the potential late effects of high-LET external beam radiotherapy (proton and ^12^C-ion) of abdominal tumors [57], which is currently not established due to the short follow-up period, and little is known about the incidence of radiation colitis among these patients.

## Supporting information

S1 File (DOCX)

## Abbreviations

IL-10: interleukin-10
CAC: Colitis associated cancer
CRC: colorectal cancer
NF-κB: nuclear factor kappa-light-chain-enhancer of activated B cells
COX2: cyclooxygenase-2
iNOS: inducible nitric oxide synthase
APC: adenomatous polyposis coli
LET: linear energy transfer
IR: ionizing radiation
