# Supplementary material for: Heavy-ion radiation-induced colitis and colorectal carcinogenesis in Il10-/- mice display co-activation of β-catenin and NF-κB signaling
Source: PLoS One. 2022 Dec 30;17(12):e0279771. doi: 10.1371/journal.pone.0279771 (PMC9803147; doi:10.1371/journal.pone.0279771)
Supplement: S1 File — (DOCX) [file pone.0279771.s001.docx]

**
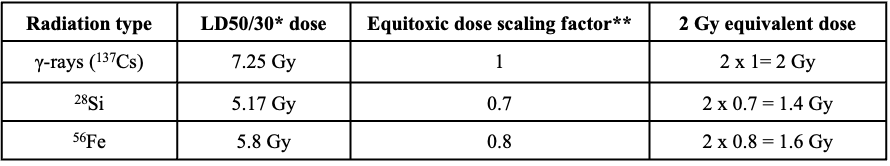
Table S1**: Calculation of 2 Gy equivalent equitoxic dose**^1-4^** for ^28^Si and ^56^Fe ions.

*As reported in references 1-2 below.

**Ratio of (^28^Si or ^56^Fe LD50/30) and 7.25 (γ-rays LD50/30) dose.

1. Datta K, Suman S, Trani D, Doiron K, Rotolo JA, Kallakury BV, Kolesnick R, Cole MF, Fornace AJ Jr. Accelerated hematopoietic toxicity by high energy (56) Fe radiation. Int J Radiat Biol. 2012 Mar;88(3):213-22. PMID: 22077279
2. Suman S, Datta K, Trani D, Laiakis EC, Strawn SJ, Fornace AJ Jr.Relative biological effectiveness of 12C and 28Si radiation in C57BL/6J mice. Radiat Environ Biophys. 2012 Aug;51(3):303-9. PMID: 22562428
3. Datta K, Suman S, Kallakury BV, Fornace AJ Jr. Exposure to heavy ion radiation induces persistent oxidative stress in mouse intestine. PLoS One. 2012;7(8): e42224. PMID: 22936983
4. Datta K, Suman S, Kallakury BV, Fornace AJ Jr. Heavy ion radiation exposure triggered higher intestinal tumor frequency and greater β-catenin activation than γ radiation in APC(Min/+) mice. PLoS One. 2013;8(3): e59295. PMID: 23555653
